# Temperature-Dependent Optical Properties of Oxidized Graphenes

**DOI:** 10.3390/nano13152263

**Published:** 2023-08-07

**Authors:** Talia Tene, Paola G. Vinueza-Naranjo, Yesenia Cevallos, Fabian Arias Arias, Matteo La Pietra, Andrea Scarcello, Yolenny Cruz Salazar, Melvin Arias Polanco, Salvatore Straface, Cristian Vacacela Gomez, Lorenzo S. Caputi, Stefano Bellucci

**Affiliations:** 1 Department of Chemistry, Universidad Técnica Particular de Loja, Loja 110160, Ecuador; 2 College of Engineering, Universidad Nacional de Chimborazo, Riobamba 060108, Ecuador; 3 Diego de Robles y Vía Interoceánica, Universidad San Francisco de Quito, Quito 170901, Ecuador; 4 Facultad de Ciencias, Escuela Superior Politécnica de Chimborazo (ESPOCH), Riobamba 060155, Ecuador; 5 INFN—Laboratori Nazionali di Frascati, 00044 Frascati, Italy; 6 Department of Information Engineering, Polytechnic University of Marche, 60131 Ancona, Italy; 7 UNICARIBE Research Center, University of Calabria, 87036 Rende, Italy; 8 Surface Nanoscience Group, Department of Physics, University of Calabria, Via P. Bucci, Cubo 33C, 87036 Rende, Italy; 9 Instituto Tecnológico de Santo Domingo, Área de Ciencias Básicas y Ambientales, Av. Los Próceres, Santo Domingo 10602, Dominican Republic; 10 Department of Environmental Engineering (DIAm), University of Calabria, Via P. Bucci, Cubo 42B, 87036 Rende, Italy

**Keywords:** graphene oxide, reduced graphene oxide, citric acid, optical bandgap, absorption coefficient

## Abstract

In this study, we investigate how changing important synthesis-related parameters can affect and control the optical characteristics of graphene oxide (GO) and reduced graphene oxide (rGO). These parameters include drying time and reduction time at two different temperatures. We obtain an understanding of their impact on optical transitions, optical bandgap, absorption coefficient, and absorbance spectrum width by analyzing these factors. Accordingly, GO has an optical bandgap of about 4 eV, which is decreased by the reduction process to 1.9 eV. Both GO and rGO display greater absorption in the visible spectrum, which improves photon capture and boosts efficiency in energy conversion applications. Additionally, our results show that GO and rGO have higher absorption coefficients than those previously reported for dispersions of exfoliated graphene. Defects in GO and rGO, as well as the presence of functional oxygen groups, are the main contributors to this increased absorption. Several measurements are carried out, including spectroscopic and morphological studies, to further support our findings.

## 1. Introduction

The basic building block of carbon-based materials such as graphite is called graphene, which is made up of a single layer of carbon atoms organized in a hexagonal lattice [1]. The remarkable physical and chemical characteristics of this two-dimensional (2D) material include exceptional electrical and thermal conductivity, mechanical strength, and a large surface area [2]. However, there are some difficulties related to graphene. For instance, it cannot be further processed because it is insoluble in water and the majority of organic solvents [3]. Graphene also has a zero bandgap, which makes it act more as a semimetal than a semiconductor. The functional groups that might be advantageous for bioapplications are also absent [4].

In this context, it has suggested derivatives such as graphene oxide (GO) and reduced graphene oxide (rGO) to get around the drawbacks of pure graphene [5]. Graphene is given oxygen-containing functional groups, such as hydroxyl (-OH), epoxy (-O-), and carboxyl (-COOH) groups, to produce GO [6]. With this change, GO becomes hydrophilic and is easier to manipulate. Additionally, GO develops a finite bandgap due to the presence of oxygen functional groups, which turns it into a semiconductor or insulating material [7]. However, reducing GO results in the production of rGO, which partially restores the electrical and mechanical properties of graphene due to the removal of oxygen functional groups [8]. Both GO and rGO have unique features and have potential in a range of applications, such as energy storage, water treatment, biosensors, composites, and catalysis [9,10].

On the other hand, the physical and chemical characteristics of GO and rGO are significantly influenced by the oxidation method [11,12,13] as well as the reducing agent (e.g., hydrazine, ascorbic acid, and citric acid [14,15,16]). Indeed, there are different levels of oxidation or reduction as a result of these processes. A thorough investigation of crucial factors such as temperature and contact time with the reducing agent is still lacking, although much research has been devoted to the synthesis processes and reducing agents for synthesizing GO and rGO. There has recently been a focus on comprehending the crucial parameters, such as drying time and reduction time, that enable control over oxygen functionalization [17]. This is crucial because it may speed up the preparation of GO and rGO with bandgaps that are comparable to those of silicon, making them ideal for next-generation devices. [18]

In a previous study conducted by Kumar et al. [19], a straightforward thermal annealing technique was developed, eliminating the need for any chemical treatments to modify as-made GO. This method successfully converts the mixed sp^2^–sp^3^ hybridized GO phases into distinct oxidized and graphitic phases during annealing, resulting in significant improvements and a noticeable enhancement in visible absorption characteristics. However, the study does not specifically address the optical bandgap, electronic transitions, or absorption coefficient of the material. Also, the study does not cover the manipulation of rGO. Therefore, further exploration is necessary to fully understand the potential of this strategy in manipulating the properties of both GO and rGO.

In the present work, we focus on examining the influence of drying time and low-temperature reduction treatment on the optical properties of various GO and rGO samples. The optical characteristics are investigated using UV-visible spectroscopy by the absorbance spectra and Tauc analysis [20]. In addition, energy-dispersive X-ray spectroscopy (EDS), X-ray powder diffraction (XRD), and scanning electron microscopy (SEM) techniques are employed to analyze the elemental composition, crystallinity, and morphology of the materials. The results indicate that increasing the drying time up to 120 h at 80 °C leads to a modification of the optical bandgap of GO from approximately 4 to 2.8 eV. Similarly, the reduction of GO-using citric acid (CA) at 80 °C for 120 h adjusts the optical bandgap of rGO from approximately 2.5 to 1.9 eV. Additionally, this study discusses in detail the estimated values of the optical absorption coefficient, shifts in electronic transitions, and relevant photosensitive characteristics.

## 2. Material and Methods

**Figure 1:**
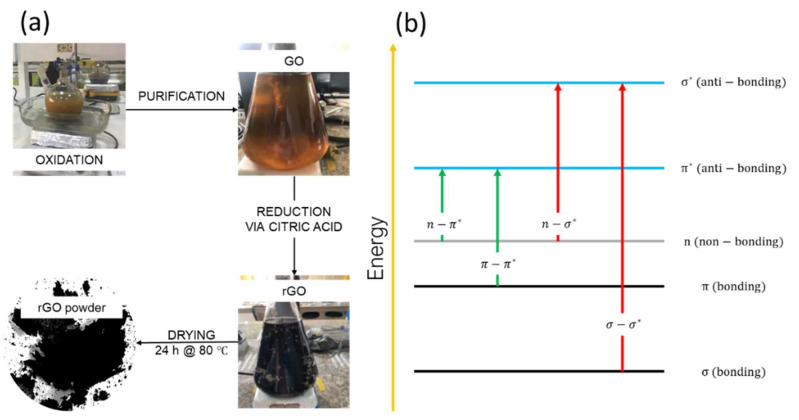
(**a**) Schematic illustration of the method used to prepare GO and rGO. (**b**) UV-visible theory.

Figure 1a summarizes the synthesis of GO and rGO. The reader is advised to study our earlier work [21] for a more thorough description of the entire procedure. The uses of GO and rGO in the elimination of pollutants are also extensively covered in our earlier research [22,23].

### 2.1. Materials

Without further purification, we used all chemical compounds exactly as they were given to us. Graphite powder (<150 μm, 99.9%) (Sigma Aldrich, St. Louis, MO, USA), sulfuric acid (H_2_SO_4_, ACS reagent, 95.0–98.0%) (Sigma Aldrich), potassium permanganate (KMnO_4_, ACS reagent, 99.0%) (Sigma Aldrich), hydrochloric acid (HCl, ACS reagent, 37%) (Sigma Aldrich), citric acid (CA, C_6_H_8_O_7_, ≥99.5%) (Sigma Aldrich), and hydrogen peroxide (H_2_O_2_, 30%) (Merk, Rahway, NJ, USA).

### 2.2. Synthesis of GO and rGO

To prepare the mixture (Figure 1a), 3.0 g of graphite powder was combined with 70 mL of H_2_SO_4_ in an ice bath. Then, 9 g of KMnO_4_ was added while keeping the temperature below 20 °C. After 0.5 h, the mixture was transferred to a water bath and stirred at 50 °C for 0.5 h. Gradually, 150 mL of distilled water was added to the solution, ensuring the temperature stayed below 90 °C. An additional 500 mL of distilled water and 15 mL of H_2_O_2_ were added. After 1 h, the resulting mixture was divided into centrifuge tubes and washed with a 1:10 solution of HCl and distilled water using several centrifugations at 10,000 rpm for 10 min. The obtained precipitate was dried at 45 °C for 48 h in a Teflon container to obtain graphite oxide powder.

In a typical experiment, 100 mg of graphite oxide powder was sonicated for 0.5 h in 500 mL of distilled water. The suspension was then centrifuged at 500 rpm for 10 min to obtain a homogeneous GO suspension. This suspension was dried at 80 °C for varying drying times ranging from 0 h to 120 h.

Following the drying process, under vigorous stirring, 500 mg of CA was gradually added to a 250 mL GO aqueous solution (1:1). Different reduction times from 1 h to 120 h were tested at 50 °C and 80 °C. To remove excess CA, the resulting black precipitates were washed with distilled water through centrifugation at 3000 rpm for 0.5 h. Finally, the precipitated material was dried at 80 °C for 24 h to obtain rGO powder.

We encountered a technical limitation when operating at temperatures exceeding 80 °C. Given that water serves as the primary medium for the synthesis process involving oxidation and reduction, evaporation becomes a significant concern, leading to a reduction in the initial water volume. Consequently, for example, at 100 °C, we must replenish the water to maintain a constant supply throughout the entire 120 h duration. However, this adjustment directly affects the initial concentration of GO, thereby impacting the resulting absorbance spectra. To mitigate this challenge and promote a more uniform procedure, we deliberately selected these two temperatures, i.e., 50 °C and 80 °C.

### 2.3. Characterization

The absorption spectra of GO and rGO were recorded using a Jenway 6850 spectrophotometer with a resolution of 0.1 nm in a wavelength window from 190 to 1000 nm. After the specified drying time or reduction time, the obtained samples were sonicated briefly to redisperse GO or rGO before measuring their absorbance spectra. The optical absorption coefficient was obtained by setting λ=660 nm. Quartz cuvettes (3.5 mL) with a 10 mm optical path were used. Spectra were normalized to the maximum of the prominent peak and conventional Lorentz functions were used to fit the curve. Additionally, absorbance spectra were smoothed using a 7-point moving average.

Using a scanning electron microscope (SEM, JSM-IT100 InTouchScope, JEOL, Tokyo, Japan) with an accelerating voltage of 20 kV and tailored with an energy-dispersive X-ray spectrometer (EDS), the surface morphology of the acquired samples was examined. Using a Panalytical Pro X-ray diffractometer with Cu K irradiation with an acceleration voltage of 60 kV and a current of 55 mA, measurements of X-ray diffraction (XRD) were made.

## 3. Results and Discussion

To emphasize even more, our team [23] and others [24,25] have successfully shown how to convert GO into rGO utilizing CA as a less dangerous and more environmentally friendly reducing agent. Due to the potential health dangers connected with hydrazine, CA is a preferable choice even though its reduction efficiency is not as high as that of hydrazine. As a result, the findings of this study can be utilized to compare and extrapolate the effectiveness of different green or non-green reducing agents.

### 3.1. Optical Properties of GO

**Figure 2:**
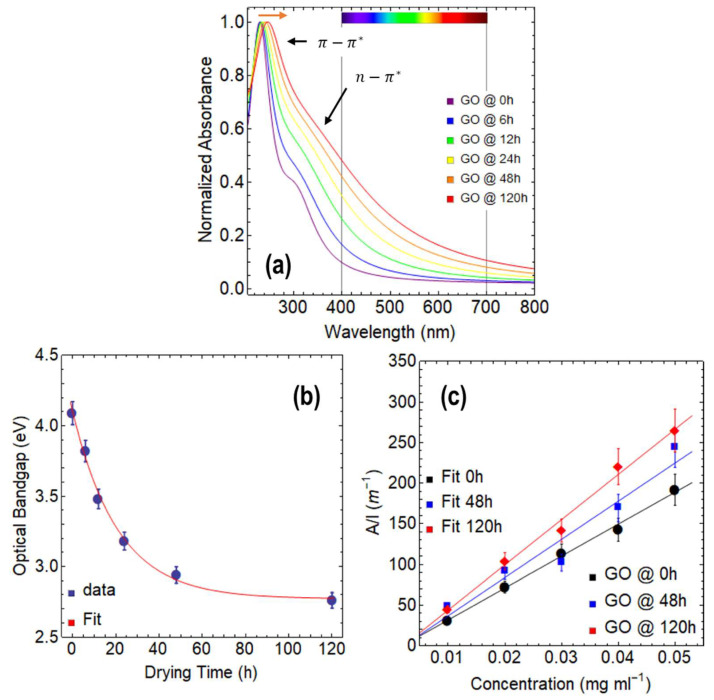
(**a**) Absorbance spectra of GO for different drying times at 80 ℃ from 200 to 800 nm. (**b**) Optical bandgap of GO as a function of drying time. (**c**) Optical absorbance at 660 nm as a function of concentration, considering three drying times. The color bar in (**a**) represents the visible region of the spectrum, spanning from 400 to 700 nm.

To facilitate an effective comparison, our study initially focused on analyzing the properties of GO under varying drying times. Figure 2a (Figure S2) displays the absorption spectra of GO with drying times of up to 120 h. The spectrum reveals two distinct absorption peaks: one at approximately 230 nm and the other at 300 nm. Based on the UV-visible theory (Figure 1b), these peaks correspond to the $\pi -\pi^{*}$ transition of C=C in amorphous carbon systems, and a broad $n-\pi^{*}$ transition of C=O bonds, respectively. All spectra are featureless in the visible region.

Notably, a redshift in the primary absorption peak is observed (Figure S1) from 229.6 nm at 0 h to 243.8 nm at 120 h (Table S1), which was not previously reported by Kumar [19]. This finding is intriguing since the evaporation of surface water molecules and water molecules within the structure of GO is expected to occur during the drying process. Such evaporation would result in a decrease in the number of functional groups and a partial reduction of the material, which could explain the observed redshift.

Our study confirms that increasing the drying time leads to a significant improvement in absorption in the visible region (Figure 1a, the color bar from 400 to 700 nm), which is a crucial factor for many applications, such as solar cells. Specifically, an improvement in photon collection is observed of almost 40% at 400 nm and 15% at 700 nm when the drying time of GO is increased. This result implies that the optical properties of GO can be tuned by adjusting the drying time, which could have significant implications for the design and optimization of various optoelectronic devices.

The Tauc approach is a common method used to estimate the optical bandgap of GO [26,27]. The Tauc approach involves plotting the absorption coefficient (∝) against photon energy and extrapolating the linear portion of the curve to the point where it intersects the x-axis. This enables the estimation of the optical bandgap, which is associated with the energy of photons that can be absorbed by the material.

With this in mind, the optical bandgap of the GO samples was estimated by fitting the linear portion between 4 eV and 5 eV for each drying time (Figure S3). It is worth noting that the optical bandgap of GO at 0 h is located outside the visible region, but as the drying time increases, the bandgap energy decreases and enters the visible region.

The numerical values for the optical bandgap can be observed in Figure 2b and Table S2. As the drying time increases, a decreasing exponential behavior is observed, with the bandgap reducing from 4.1 eV at 0 h to 2.8 eV at 120 h. After fitting the data, we obtained the equation: $y=1.33\;\mathrm{Exp}\left[-4.77\times 10^{-2}\;t\right]+2.77$ ($R^{2}=0.999$), which implies that the effect of drying time on the optical bandgap diminishes after 120 h, as the maximum predicted reduction in optical bandgap is 2.77 eV. This result is further supported by the small rate of decrease observed, which is $-4.77\times 10^{-2}$ s^−1^. The largest impact of drying time is observed between 0 and 24 h, resulting in a decrease of 0.91 eV.

To calculate the absorption coefficient, we followed the Lambert–Beer law ($A=\alpha_{660}\cdot c\cdot l$) and prepared multiple GO dispersions with varying concentrations ranging from 0.01 to 0.05 mg mL^−1^. This method is commonly used for exfoliated graphene dispersions in water or alcohols [28,29] and has now been applied to GO samples at different drying times. The linear relationship between concentration (c) and absorbance (A), which is independent of drying time, is confirmed by Figure 2c. Additionally, it can be observed that the ratio of A/l increases as the drying time increases, indicating a rise in the absorption coefficient. The absorption coefficient of GO at different drying times was found to be 3932.22 mL mg^−1^ m^−1^ at 0 h, 4586.71 mL mg^−1^ m^−1^ at 48 h, and 5507.15 mL mg^−1^ m^−1^ at 120 h (Table S3). These values are significantly higher than those reported for exfoliated graphene dispersions, which typically have an absorption coefficient of around 2460 mL mg^−1^ m^−1^ [28].

The higher absorption coefficient of GO compared to exfoliated graphene is likely due to the presence of oxygen functional groups in its structure [30]. These functional groups create defects in the carbon lattice, which can interact with photons at a wider range of energies than exfoliated graphene. Additionally, the oxygen functional groups on the surface of GO can induce dipole moments and charge transfer, further enhancing the absorption of electromagnetic radiation. It is crucial to note that as the drying time increases (from 0 h to 120 h) (Figure 2d), the absorption coefficient also increases. This observation suggests that while the number of oxygen functional groups may decrease, a disordered and defective structure might prevail due to the increase in the number of vacancies [30].

With these results in mind, GO seems useful in applications such as photovoltaics, photocatalysis, and optoelectronics where high absorption coefficients are required.

On the other hand, we have noticed a quasi-linearity in the position of the main absorption peak ($\pi -\pi^{*}$ transition) as a function of drying time (Figure S4a) as well as the optical bandgap as a function of the position of the $\pi -\pi^{*}$ transition (Figure S4b). Perfect linearity is also observed between the optical bandgap and the full width at half maximum (FWHM) (Figure S4c). Beyond these interesting results, we can note something important: as the drying time increases, the FWHM significantly increases (Table S1).

### 3.2. Optical Properties of rGO Reduced at 80 °C

**Figure 3:**
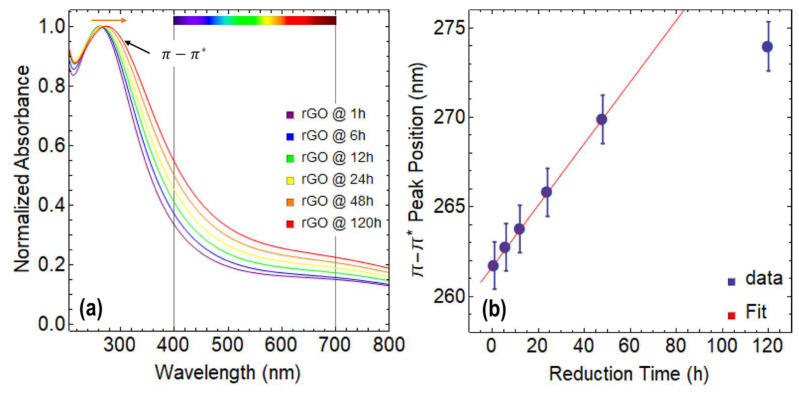
(**a**) Absorbance spectra of rGO reduced at 80 °C from 200–800 nm. (**b**) Position of $\pi -\pi^{*}$ transition as a function of reduction time. The color bar in (**a**) represents the visible region of the spectrum, spanning from 400 to 700 nm.

**Figure 4:**
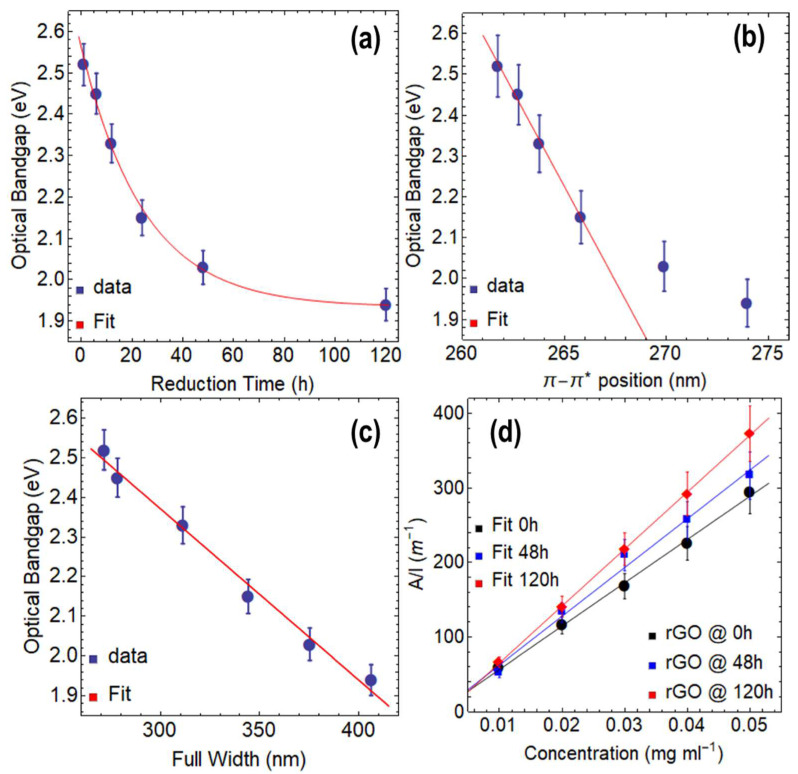
Optical bandgap of rGO reduced at 80 °C as a function of: (**a**) reduction time, (**b**) position of $\pi -\pi^{*}$ transition, and (**c**) full-width at half maximum (FWHM). (**d**) Optical absorbance at 660 nm as a function of concentration, considering three reduction times.

We now proceed to analyze the properties of rGO (Figure 3 and Figure 4). For the reduction and seeing the potential scalability of the process, we focused on the GO samples dried at 80 °C for 24 h, which showed the most efficient result of the drying process. These GO samples resulted in a bandgap of 3.2 eV (Table S2) and a $\pi -\pi^{*}$ transition at 233.3 nm (Table S1). However, it is worth considering the possibility of utilizing the samples dried for 120 h, which would result in a partially reduced material due to the thermal reduction treatment. This would undoubtedly impact the reduction time required or even the choice of reducing agent. This concept serves as a motivation for future extensive studies aimed at optimizing the process and evaluating the cost-benefit of employing a material subjected to prolonged drying time.

Figure 3a displays the absorption spectra of rGO reduced at 80 °C using CA under various reduction times ranging from 1 h to 120 h. Notably, all the spectra appear featureless in the visible region, indicating that the reduction process did not introduce any extra species (Figure S6). Additionally, it is noteworthy that only the $\pi -\pi^{*}$ transition is observed, while the $n-\pi^{*}$ transition is absent in all the spectra.

In Figure S5, a redshift of the $\pi -\pi^{*}$ transition is observed, which shifts from 261.7 nm at 1 h of reduction with CA to 273.9 nm at 120 h of reduction (Table S4). This shift is much more significant compared to the one observed in GO, indicating that the reduction process has occurred, and the structure and properties of graphene are progressively recovering.

Upon careful analysis of Figure 3a, an important rise in absorption in the visible region can be observed, with up to a 22% increase at 400 nm and a 9% increase at 700 nm as the reduction time increases. Although these results are lower than those observed for GO, the improved absorption in the visible region makes rGO a promising material with several potential applications.

On the other hand, the position of the $\pi -\pi^{*}$ transition follows a clear linear trend until 48 h of reduction time, after which it starts to plateau because the point at 120 h strongly deviates from this linearity and the position of this peak cannot increase indefinitely (Figure 3b). It is worth noting that the maximum position of the $\pi -\pi^{*}$ transition for exfoliated graphene dispersions is around 280 nm [31]. Similar to GO, the FWHM of the $\pi -\pi^{*}$ transition observed in rGO also increases as the reduction time progresses, indicating a broadening of the main absorption peak (Table S4).

We now focus on discussing the optical bandgap (Table 1 and Figure S7) and absorption coefficient (Table 2) of rGO. Figure 4a shows a decrease in the optical bandgap from 2.52 eV at 1 h of reduction to 1.94 eV at 120 h of reduction, which can be described by a decreasing exponential function ($y=0.63\;\mathrm{Exp}\left[-4.04\times 10^{-2}\;t\right]+1.93$, $R^{2}=0.999$). The total drop in the optical bandgap through the reduction process at 80 °C via CA is 0.58 eV. Compared to GO at 0 h of drying time and GO at 24 h of drying time, the optical bandgap decreases down to 2.15 eV and 1.24 eV, respectively. The most significant decrease in the optical bandgap occurs during the first 24 h of reduction, with a difference of 0.37 eV. Beyond this point, the effect of the reducing agent on the optical bandgap continues to slightly decrease with a small decreasing rate of $-4.04\times 10^{-2}$ s^−1^, leading to a minimum optical bandgap of 1.93 eV by using CA.

Figure 4b shows a linear relationship between the optical bandgap and the position of the $\pi -\pi^{*}$ transition up to 24 h of reduction time, which can be expressed by data fitting (Table 1) as $y=-9.27\times 10^{-2}\;t+26.80$ ($R^{2}=0.995$). The plot demonstrates that the data points at 48 h and 120 h of reduction do not align with the linear fit, indicating that both the optical bandgap and the position of the $\pi -\pi^{*}$ transition have reached a saturation point, and the reduction with CA has almost entirely taken place. The slope is $-9.27\times 10^{-2}$ eV nm^−1^, indicating that the reduction of the optical bandgap for each nm of the redshift of the $\pi -\pi^{*}$ transition is slow but steady.

Figure 4c shows a linear relationship between the optical bandgap and the FWHM, which can be described by the following equation: $y=-4.32\times 10^{-3}\;t+3.66$ ($R^{2}=0.989$). Although the slope is very small ($-4.32\times 10^{-3}$ eV nm^−1^), the impact of reduction time on the FWHM is significant. For instance, rGO reduced for 1 h has an optical bandgap of 2.52 eV and an FWHM of 271.57 nm, while rGO at 120 h of reduction shows an optical bandgap of 1.94 eV and an FWHM of 406.23 nm. These results further highlight the importance of controlling the reduction time to obtain rGO with desirable features.

Figure 4d shows the ratio of A/l, which exhibits a linear increase with increasing the concentration of rGO, regardless of the reduction time. By fitting the data, absorption coefficient values of 5803.89 mL mg^−1^ m^−1^ at 1 h, 6534.43 mL mg^−1^ m^−1^ at 48 h, and 7638.10 mL mg^−1^ m^−1^ at 120 h were attained (Table 2). These values are almost three times larger than those reported for exfoliated graphene dispersions (~2460 mL mg^−1^·m^−1^ [28]), indicating the superior light absorption capability of rGO. Compared to GO, the absorption coefficients of rGO show an increase of nearly 28%.

To gain a deeper understanding of these results, we propose the following idea: the superior absorption coefficient of rGO can be attributed to the reduction process, which removes oxygen functional groups and restores the sp^2^ hybridization of carbon atoms in certain regions of the graphene lattice. This restoration leads to a significant increase in the number of confined π− electrons perpendicular to the plane, thereby enhancing light absorption. This effect is well-documented in the field of graphene plasmonics [32]. Additionally, it is worth noting that the reduction process may induce defects and vacancies in certain regions of the graphene lattice, which also contribute substantially to increased light absorption [30].

### 3.3. Optical Properties of rGO Reduced at 50 °C

**Figure 5:**
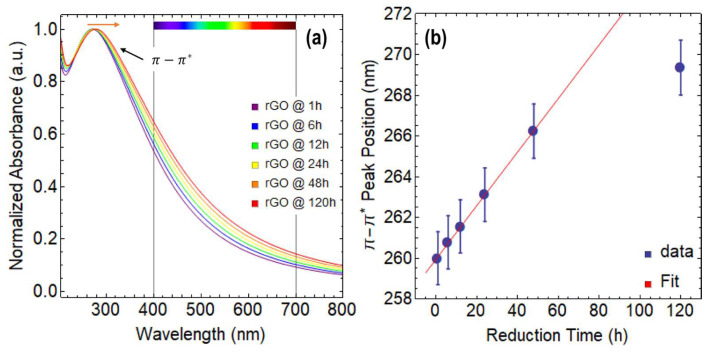
(**a**) Absorbance spectra of rGO reduced at 50 °C from 200 to 800 nm. (**b**) Position of $\pi -\pi^{*}$ transition as a function of reduction time. The color bar in (**a**) represents the visible region of the spectrum, spanning from 400 to 700 nm.

**Figure 6:**
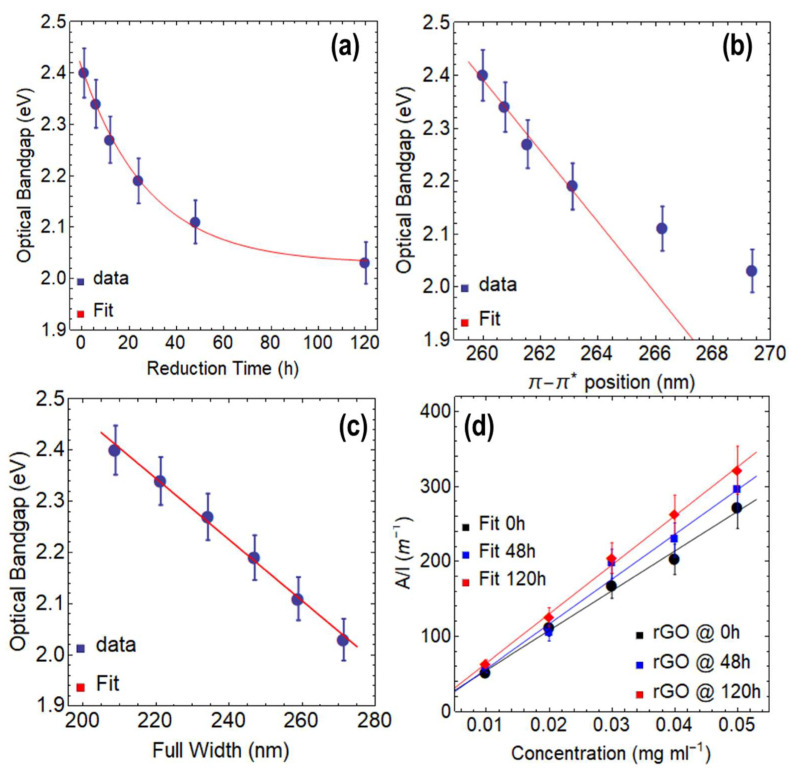
Optical bandgap of rGO reduced at 50 °C as a function of: (**a**) reduction time, (**b**) position of $\pi -\pi^{*}$ transition, and (**c**) full-width at half maximum (FWHM). (**d**) Optical absorbance at 660 nm as a function of concentration, considering three reduction times.

Similar to the previous section, we now analyze rGO reduced at 50 °C (Figure 5 and Figure 6). Figure 5a shows the absorbance spectra of rGO for different reduction times. Although the spectra do not present significant features in the visible region (Figure S9), the broad structure of the $\pi -\pi^{*}$ peak remains remarkable. On the other hand, Figure S8 shows a slight redshift from 260.33 nm at 1 h of reduction to 269.36 nm at 120 h of reduction (Table S5). This smaller displacement of the $\pi -\pi^{*}$ peak can be attributed to the fact that reduction temperature critically affects the kinetics of the reduction process.

Additionally, Figure 5a demonstrates an increase in light absorption in the visible region for rGO reduced at 50 °C, but the enhancement is significantly less when compared to GO. At 400 nm, the improvement is only around 10%, and at 700 nm, it is about 5%. Nonetheless, these findings showcase the adaptability of GO and rGO in adjusting their interaction with light by manipulating various parameters, including the oxidation-reduction processes and the reducing agents employed.

A plateau in the position of the $\pi -\pi^{*}$ peak is observed in Figure 5b at 120 h of reduction time, even at 50 °C, indicating that the peak position reaches saturation after 48 h of reduction where a clear linearity is observed from 1 h to 48 h of reduction. This behavior is similar to that observed for rGO reduced at 80 °C. Additionally, the FWHM of the absorbance spectrum also increases as the reduction time increases (Table S5) in rGO reduced at 50 °C. However, the increase is much lower compared to rGO reduced at 80 °C. As an example, at 1 h, the FWHM is 208.94 nm, and at 120 h, it is 271.41 nm.

Interestingly, when rGO is reduced at 50 °C for 1 h (Figure 6a and Figure S10a), the resulting optical bandgap is 2.40 eV (Table 3), which is a more efficient outcome than the one obtained at 80 °C for the same duration (2.52 eV). The reason for this observation is not yet clear, but it is possible that at higher temperatures, secondary reactions such as oxidation or degradation of GO occur, leading to structural defects. At lower temperatures, more stable reduction products with fewer defects may be formed. In the case of reducing GO at 50 °C, it is possible that at the lower temperature, the reducing agent used has a stronger affinity for the oxygen functional groups of GO, leading to a more efficient reduction in a shorter time. However, further research is needed to determine the specific factors that influence the efficiency of the process. Notably, after 120 h, the optical bandgap is 2.03 eV (Table 3, Figure S10f), which is higher than the value observed in the reduction of GO at 80 °C at the same duration (1.94 eV).

A good linear relationship can be observed between the optical bandgap and the position of the $\pi -\pi^{*}$ peak (Figure 6b) for up to 24 h. However, the data points at 48 h and 120 h deviate from this linear trend, indicating that a saturation point is approaching. A strong linear relationship can be observed between the optical bandgap and the FWHM (Figure 6c).

The absorbance coefficients of rGO reduced at 50 °C for various reduction times are shown in Figure 6d and Table 4. The acquired values are lower than those seen for rGO reduced at 80 °C (Table 2) but greater than those reported for dispersions of exfoliated graphene and GO (Table S3), demonstrating the major influence of the reduction temperature on the final optical characteristics of the resulting oxidized graphene derivative.

### 3.4. Spectroscopic and Morphological Measurements

**Figure 7:**
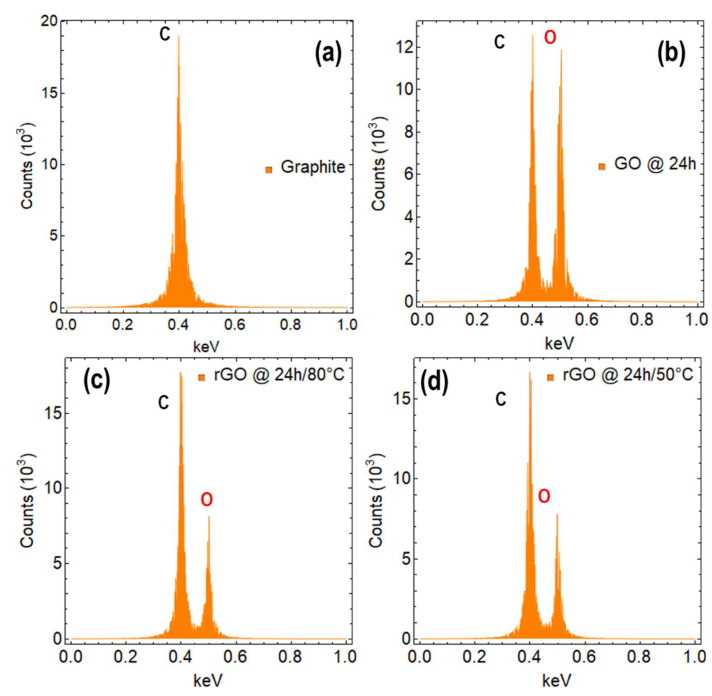
EDS measurement of (**a**) graphite, (**b**) GO dried at 80 °C for 24 h, (**c**) rGO reduced at 80 °C for 24 h, and (**d**) rGO reduced at 50 °C for 24 h. C and O denotes the chemical elements of carbon and oxygen, respectively.

**Figure 8:**
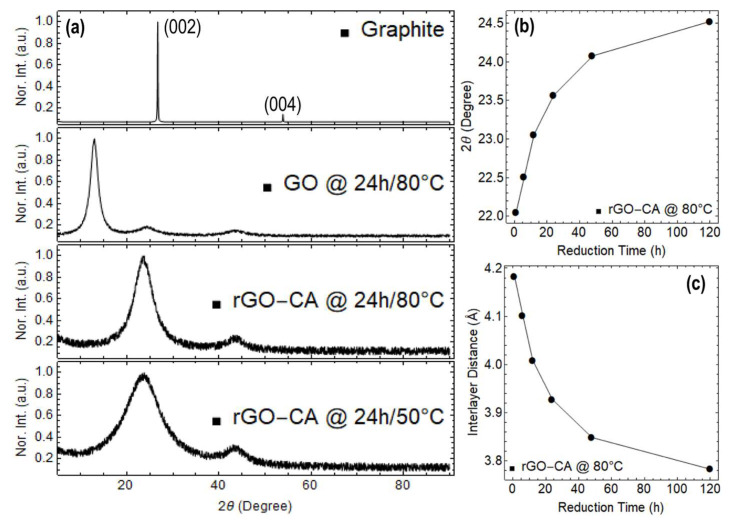
(**a**) XRD measurements on power graphite, GO, and rGO. (**b**) Variation of the 2θ peak position as a function of reduction time. (**c**) Variation of interlayer distance as a function of the reduction time. The spectra were normalized to the maximum of the prominent peak.

**Figure 9:**
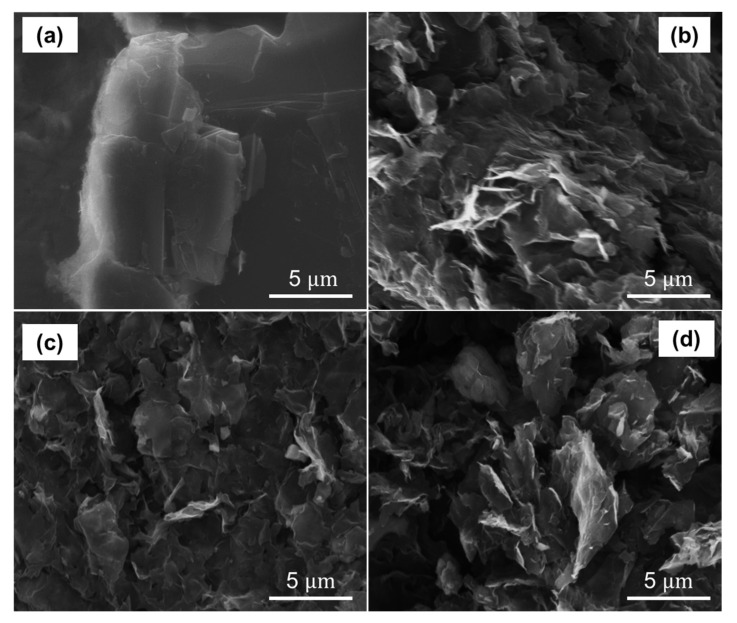
SEM measurements of (**a**) power graphite, (**b**) GO dried at 80 °C for 24 h, (**c**) rGO reduced at 80 °C for 24 h, and (**d**) rGO reduced at 50 °C for 24 h.

To clarify and support our findings, we have conducted additional measurements using EDS (Figure 7), XRD (Figure 8), and SEM (Figure 9). Notably, we placed particular emphasis on samples subjected to 24 h of drying time for GO and 24 h of reduction time for rGO at both 50 °C and 80 °C. These specific conditions were chosen to strike a balance between production time and achieving the desired optical bandgap, thereby exploring the potential for scalability as discussed in earlier sections. We would like to highlight that our EDS study encompasses a comprehensive range of drying times, spanning from 0 to 120 h, as well as different reduction times at 80 °C or 50 °C, ranging from 1 to 120 h. The corresponding results for these variations can be found in Figures S11–S13 of the Supplementary Material. Additionally, we have included the numerical data associated with these measurements in Tables S6–S8.

Figure 7a displays the EDS measurement of graphite, which presents a single peak and a mass percentage of 99.6% (Table S6). This peak confirms the presence of only carbon atoms in the sample, which is consistent with the expected composition of graphite. The mass percentage of 99.9% indicates that the sample is highly pure. Figure S11a shows the EDS measurement on GO at 0 h of drying time, where two peaks are observed for carbon and oxygen with percentage masses of 41.47% and 58.53%, respectively (Table S6). These results indicate that the sample is primarily composed of carbon and oxygen, which is expected for GO regardless of the method used for oxidation.

Figure 7b displays the EDS measurement of GO at 24 h of drying time, which reveals two peaks for carbon and oxygen, indicating the presence of both elements. The carbon peak with a percentage mass of 51.42% suggests that carbon is the major constituent of the sample, while the oxygen peak with a percentage mass of 48.58% indicates a significant amount of oxygen is present. The decrease in the percentage of oxygen from 58.53% (0 h) to 48.58% (24 h) confirms that the drying process has removed some of the oxygen functional groups from GO.

The EDS measurements on rGO at 80 °C (Figure 7c) and 50 °C (Figure 7d) for 24 h of reduction time show two peaks for carbon and oxygen, indicating the presence of both elements in the samples. The peak for carbon in both cases has a higher percentage mass, with 68.53% at 80 °C (Table S7) and 68.17% at 50 °C (Table S8), suggesting that carbon is the major constituent of the sample. Meanwhile, the peak for oxygen in both cases has a lower percentage mass, with 31.47% at 80 °C and 31.83% at 50 °C, indicating a significant amount of oxygen is still present in rGO.

These results suggest that the reduction process has not completely removed all of the oxygen functional groups from GO. However, the decrease in the percentage of oxygen confirms that the reduction process has successfully converted GO into rGO. The higher percentage of carbon in both cases of rGO compared to GO indicates that the reduction process has resulted in the restoration of some of the sp^2^ hybridized carbon bonds, which is a characteristic of graphene. Additionally, Figures S12 and S13 depict the evolution over time of the mass percentage of carbon and oxygen in rGO samples subjected to different reduction times and different temperatures. Similarly, the evolution over time of the mass percentage of carbon and oxygen in GO samples subjected to different drying times can be observed in Figure S11.

To support EDS results, Figure 8 shows the XRD measurements on the same samples. For comparison purposes, Figure 8 shows XRD measurements of graphite, which displays the (002) peak at 26.73° and the (004) peak at 55.17°, indicating the high crystalline structure of graphite. In particular, the (002) peak corresponds to the d-spacing between adjacent carbon planes in the graphite structure, which is approximately 3.35 Å.

The XRD measurement of GO dried at 80 °C for 24 h exhibits a single peak at 12.95°, indicating the presence of an interlayer spacing distance between adjacent GO layers of 6.12 Å. This value is significantly larger than the interlayer spacing distance of graphite (3.35 Å) and can be attributed to the presence of intercalated oxygen functional groups. Furthermore, this single peak in the XRD pattern suggests that GO has a disordered structure.

For rGO, a relatively narrow peak is observed at 23.57° in rGO reduced at 80 °C, indicating a recovered highly ordered structure with a narrow distribution of interlayer spacing distances between adjacent graphene sheets. On the other hand, the broad peak observed at 23.38° in rGO reduced at 50 °C suggests a less ordered structure with a wider distribution of interlayer spacing distances.

The variation of the 2θ peak position and interlayer distance in rGO as a function of reduction are shown in Figure 8b,c, respectively. In particular, the values of interlayer distance ranging from 4.18 to 3.78 Å are significantly smaller than the interlayer spacing distance in GO and can be attributed in fact to the reduction of oxygen functional groups and the restoration of the sp^2^ hybridization.

The XRD results are complemented by SEM measurements, as shown in Figure 9. The SEM image of graphite (Figure 9a) displays a regular and layered structure. Conversely, GO dried for 24 h at 80 °C exhibits a distinct structure, characterized by numerous folds in the plane and on the edges (Figure 9b). The surface is also corrugated and lacks a homogeneous structure.

Instead, rGO reduced at 80 °C for 24 h (Figure 9c) exhibits a relatively regular structure with folded edges and a moderate degree of surface roughness. In contrast, rGO reduced at 50 °C (Figure 9d) displays a stacked and disordered structure with numerous folds on the edges, but also with a relatively smooth surface. Notably, rGO reduced at 50 °C exhibits a micro- and mesoporous structure, which may be attributed to the presence of residual oxygen functional groups and defects resulting from the reduction process.

## 4. Conclusions

In conclusion, we have carefully studied the optical characteristics of GO and rGO by considering various oxidation–reduction process parameters, such as drying time, reduction time, temperature, and reduction by employing CA as a green reducing agent. The key findings of our study are summarized below:

- According to our research, GO has an optical bandgap of around 4 eV at 0 h of drying time, which gradually drops to 2.77 eV after 120 h. On the other side, rGO shows a bandgap reduction with longer reduction times. After 120 h of reduction time, the optical bandgaps of rGO at 80 °C and 50 °C were 1.94 eV and 2.03 eV, respectively.
- The $\pi -\pi^{*}$ transition predominately defines the absorbance spectra of both GO and rGO. Significantly, the aforementioned oxidation–reduction process parameters have a strong influence on this transition. For instance, the $\pi -\pi^{*}$ transition in GO occurs at about 230 nm at 0 h of drying time. On the other hand, regardless of the temperature at which the reduction is carried out, these transitions occur for rGO with only 1 h of reduction at wavelengths larger than 260 nm.
- Our study further validates that extending the drying time yields a notable enhancement in absorption within the visible region. Specifically, we observed a substantial improvement in photon collection of approximately 40% at 400 nm and 15% at 700 nm when increasing the drying time of GO. Similarly, in the case of rGO reduced at 80 °C, we observed a significant increase in absorption within the visible region, with up to a 22% rise at 400 nm and a 9% increase at 700 nm as the reduction time is extended.
- We measured high absorption coefficients in both GO and rGO, surpassing those reported for exfoliated graphene dispersions by two to three times. These findings confirm the superior optical properties of oxidized graphenes, highlighting their improved capacity for absorbing light.

## Figures and Tables

**Table 1:** Estimated optical bandgaps of rGO reduced at 80 °C under different reduction times ranging from 0 to 120 h.

Reduction Time (h)	Optical Bandgap (eV)	*R* ^2^
1	2.52	0.999
6	2.45	0.999
12	2.33	0.999
24	2.15	0.999
48	2.03	0.999
120	1.94	0.999

**Table 2:** The estimated optical absorption coefficient of rGO reduced at 80 °C, considering three reduction times.

Material	Absorption Coefficient (mL mg^−1^ m^−1^)	*R* ^2^
rGO @ 1 h/80 °C	5803.89	0.997
rGO @ 48 h/80 °C	6534.43	0.989
rGO @ 120 h/80 °C	7638.10	0.989

**Table 3:** Estimated optical bandgaps of rGO reduced at 50 °C under different reduction times ranging from 0 to 120 h.

Reduction Time (h)	Optical Bandgap (eV)	*R* ^2^
1	2.40	0.999
6	2.34	0.999
12	2.27	0.999
24	2.19	0.999
48	2.11	0.999
120	2.03	0.999

**Table 4:** The estimated optical absorption coefficient of rGO reduced at 50 °C, considering three reduction times.

Material	Absorption Coefficient (mL mg^−1^ m^−1^)	*R* ^2^
rGO @ 1 h/50 °C	5294.11	0.993
rGO @ 48 h/50 °C	5975.30	0.983
rGO @ 120 h/50 °C	6540.53	0.999

## Data Availability

Not applicable.

## Supplementary Materials

The supporting information can be downloaded at: https://www.mdpi.com/article/10.3390/nano13152263/s1.
