# Supplementary material for: Temperature-Dependent Optical Properties of Oxidized Graphenes
Source: Nanomaterials (Basel). 2023 Aug 7;13(15):2263. doi: 10.3390/nano13152263 (PMC10421430; doi:10.3390/nano13152263)
Supplement: Supplementary file 1 [file nanomaterials-13-02263-s001.zip › nanomaterials-2478938-supplementary.pdf]

Article

Temperature-Dependent Optical Properties of Oxidized Graphenes

Talia Tene [1,*], Paola G. Vinueza-Naranjo [2], Yesenia Cevallos [2,3], Fabian Arias Arias [4], Matteo La Pietra [5,6], Andrea Scarcello [7,8], Yolenny Cruz Salazar [7,8], Melvin Arias Polanco [9], Salvatore Straface [10], Cristian Vacacela Gomez [5,*], Lorenzo S. Caputi [7,8] and Stefano Bellucci [5,*]

Department of Chemistry, Universidad Técnica Particular de Loja, Loja 110160, Ecuador

[2] College of Engineering, Universidad Nacional de Chimborazo, Riobamba 060108, Ecuador; paolag.vinueza@unach.edu.ec (P.G.V.-N.); ycevallos@unach.edu.ec (Y.C.)

[3] Diego de Robles y Vía Interoceánica, Universidad San Francisco de Quito, Quito 170901, Ecuador

[4] Facultad de Ciencias, Escuela Superior Politécnica de Chimborazo (ESPOCH), Riobamba 060155, Ecuador; fabian.arias@espoch.edu.ec

[5] INFN—Laboratori Nazionali di Frascati, 00044 Frascati, Italy; matteo.lapietra@lnf.infn.it

[6] Department of Information Engineering, Polytechnic University of Marche, 60131 Ancona, Italy

[7] UNICARIBE Research Center, University of Calabria, 87036 Rende, Italy; andrea.scarcello@unical.it (A.S.); yolennymabelcruz@gmail.com (Y.C.S.); lorenzo.caputi@fis.unical.it (L.S.C.)

[8] Surface Nanoscience Group, Department of Physics, University of Calabria, Via P. Bucci, Cubo 33C, 87036 Rende, Italy

[9] Instituto Tecnológico de Santo Domingo, Área de Ciencias Básicas y Ambientales, Av. Los Próceres, Santo Domingo 10602, Dominican Republic; melvin.arias@intec.edu.do

[10] Department of Environmental Engineering (DIAm), University of Calabria, Via P. Bucci, Cubo 42B, 87036 Rende, Italy; salvatore.straface@unical.it

* Correspondence: tbtene@utpl.edu.ec (T.T.); cristianisaac.vacacelagomez@fis.unical.it (C.V.G.); bellucci@lnf.infn.it (S.B.)

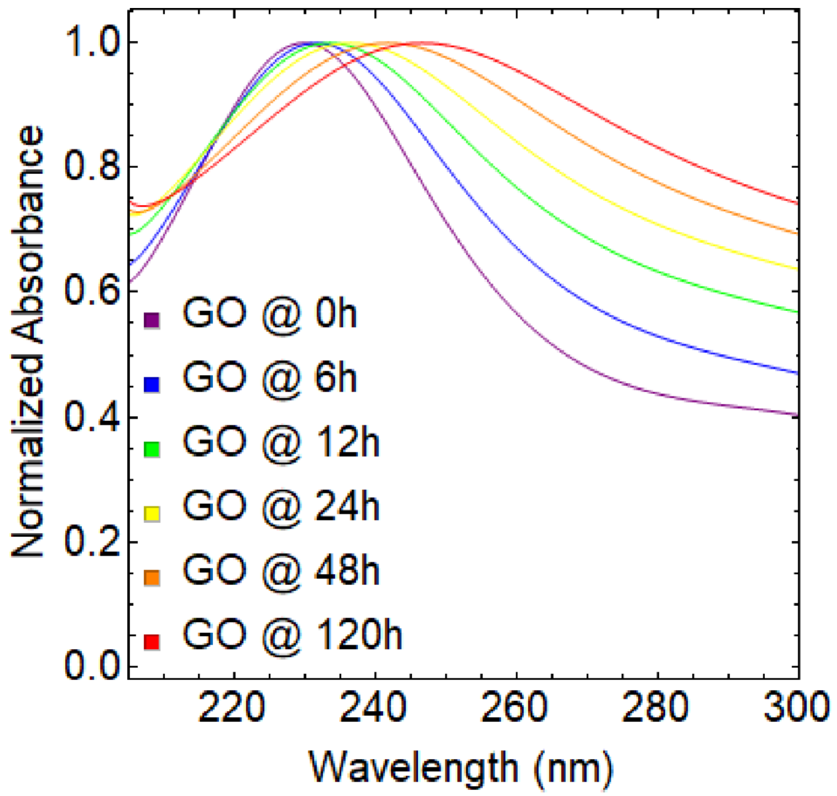

Figure S1. Absorbance spectra from 200 to 300 nm of GO dried at 80 °C and considering different drying times.

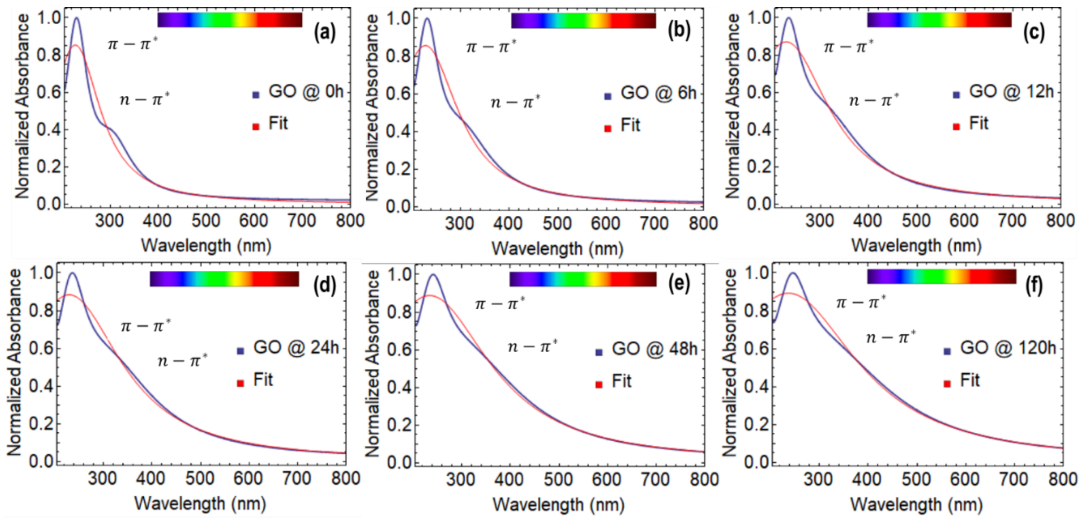

Figure S2. Absorbance spectrum of GO at 80 °C considering different drying times: (a) 0h, (b) 6h, (c) 12 h, (d) 24h, (e) 48, and (f) 120h.

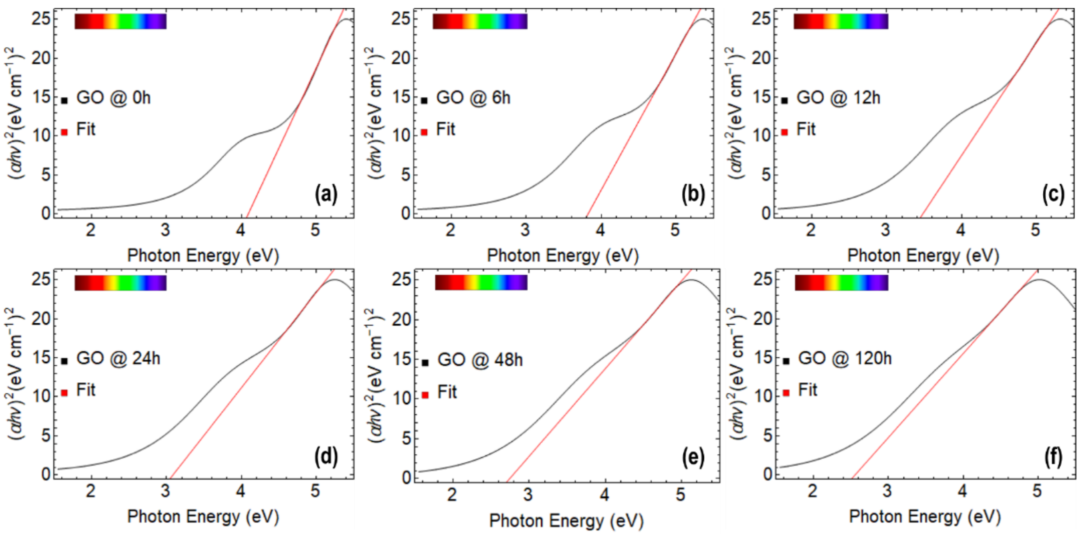

Figure S3. Tauc plot of GO at 80 ℃ considering different drying times: (a) 0h, (b) 6h, (c) 12 h, (d) 24h, (e) 48, and (f) 120h.

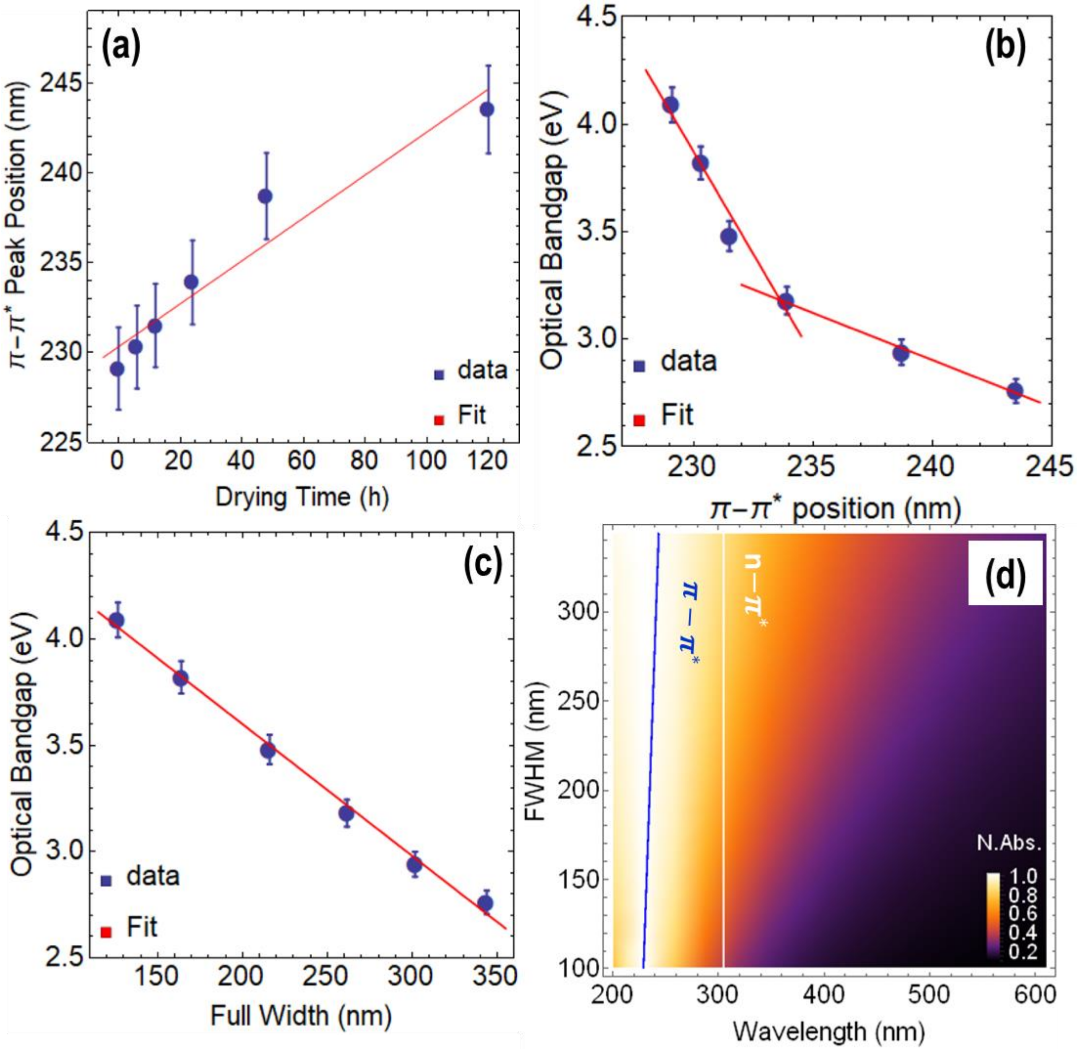

Figure S4. GO dried at 80 ℃. (a) Position of $\pi - \pi^*$ transition as a function of drying time. Optical bandgap as a function of: (b) position of $\pi - \pi^*$ transition and (c) full-width at half maximum (FWHM). (d) Normalized absorbance as a function of FWHM vs. wavelength

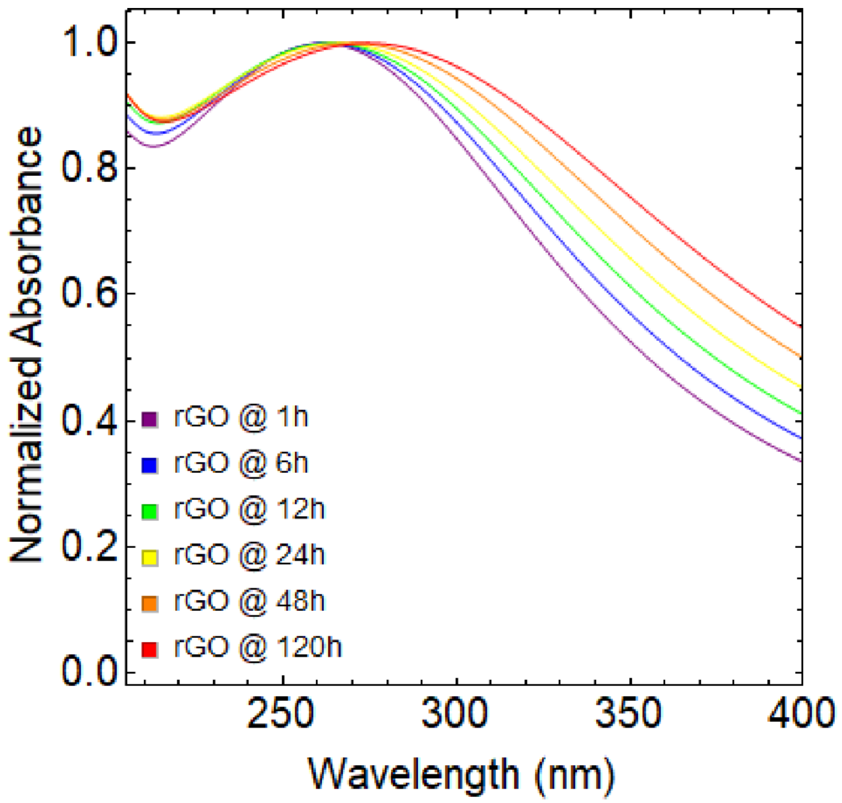

Figure S5. Absorbance spectra from 200 to 400 nm of rGO reduced at 80 °C and considering different reduction times.

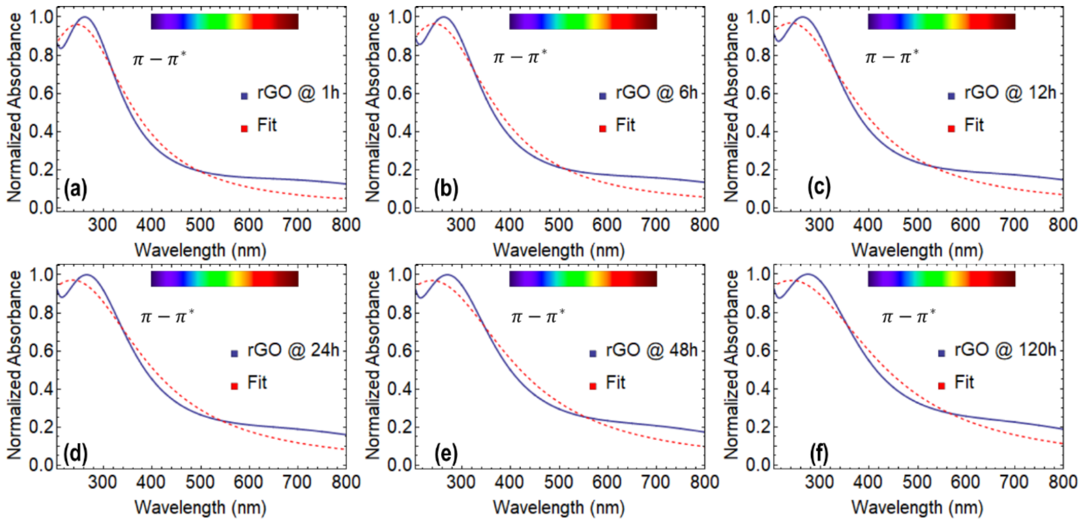

Figure S6. Absorbance spectrum of rGO reduced at 80 °C considering different reduction times: (a) 0h, (b) 6h, (c) 12 h, (d) 24h, (e) 48, and (f) 120h.

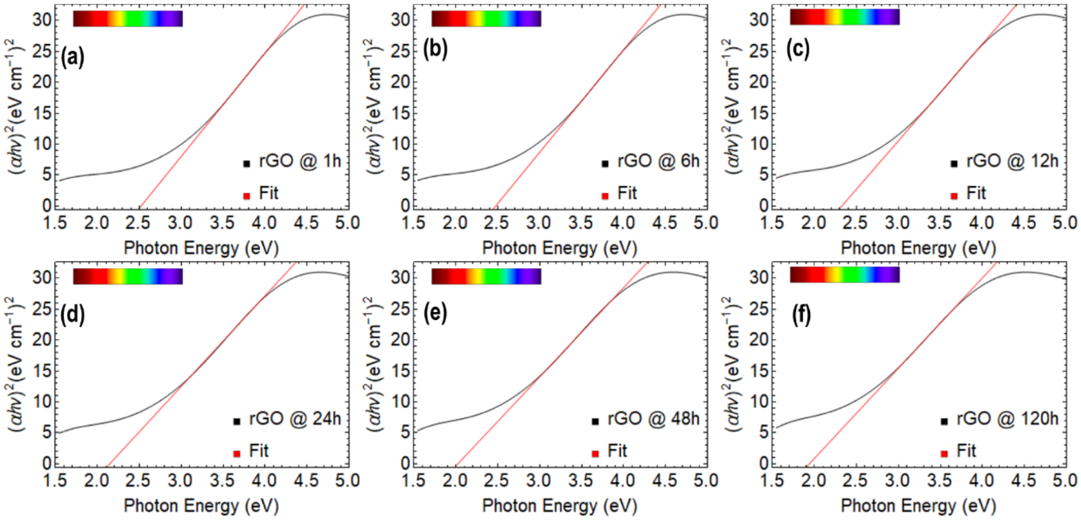

Figure S7. Tauc plot of rGO reduced at 80 ℃ considering different reduction times: (a) 0h, (b) 6h, (c) 12 h, (d) 24h, (e) 48, and (f) 120h.

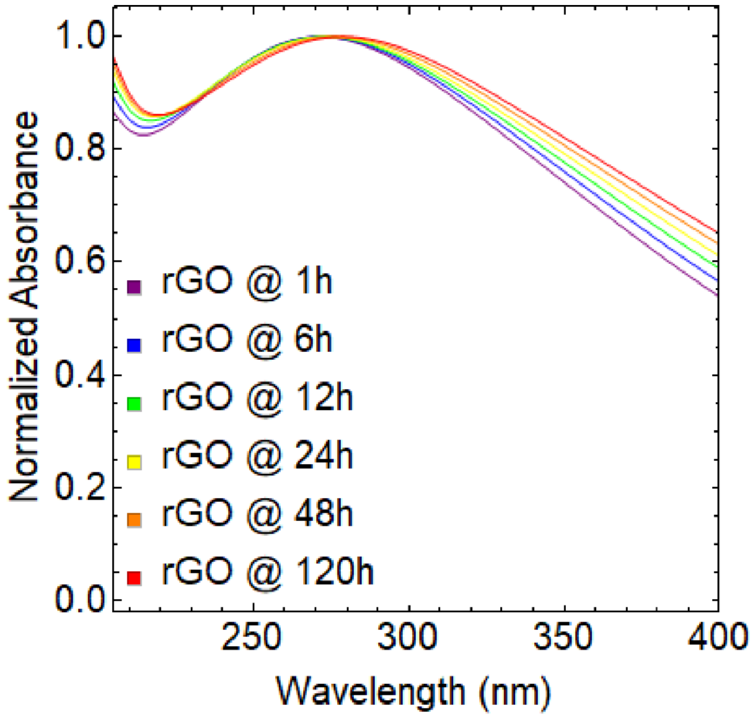

Figure S8. Absorbance spectra from 200 to 400 nm of rGO reduced at 50 ℃ and considering different reduction times.

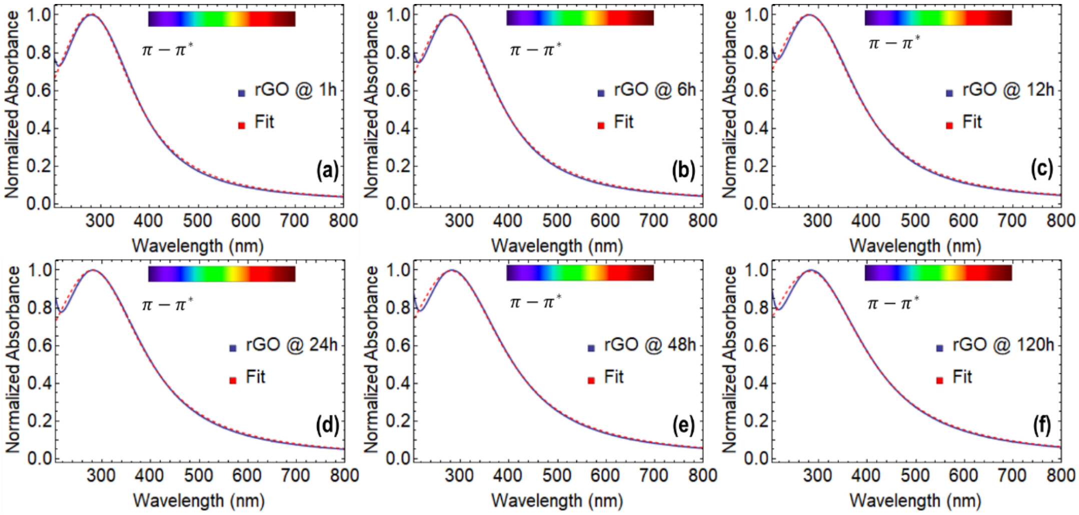

Figure S9. Absorbance spectrum of rGO reduced at 50 ℃ considering different reduction times: (a) 0h, (b) 6h, (c) 12 h, (d) 24h, (e) 48, and (f) 120h.

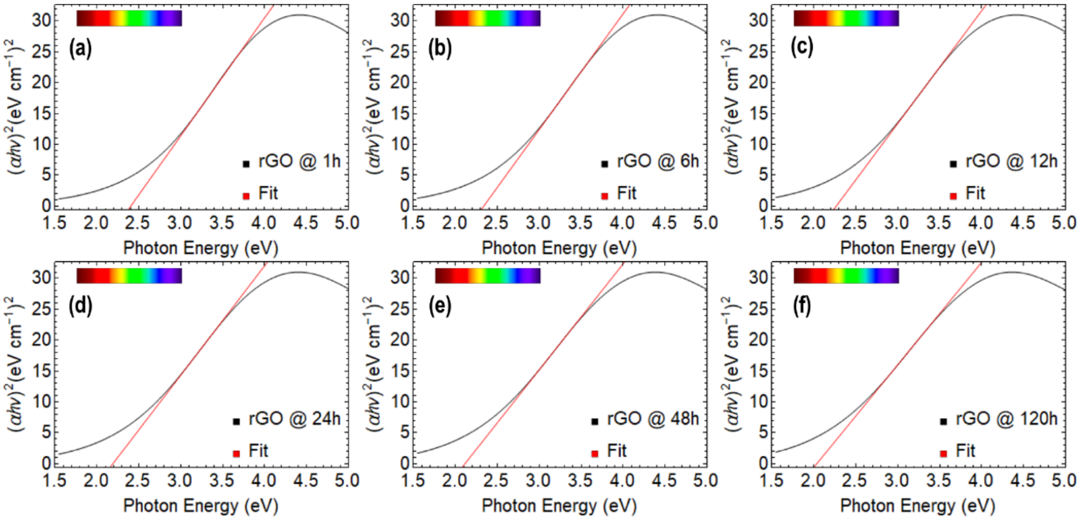

Figure S10. Tauc plot of rGO reduced at 50 ℃ considering different reduction times: (a) 0h, (b) 6h, (c) 12 h, (d) 24h, (e) 48, and (f) 120h

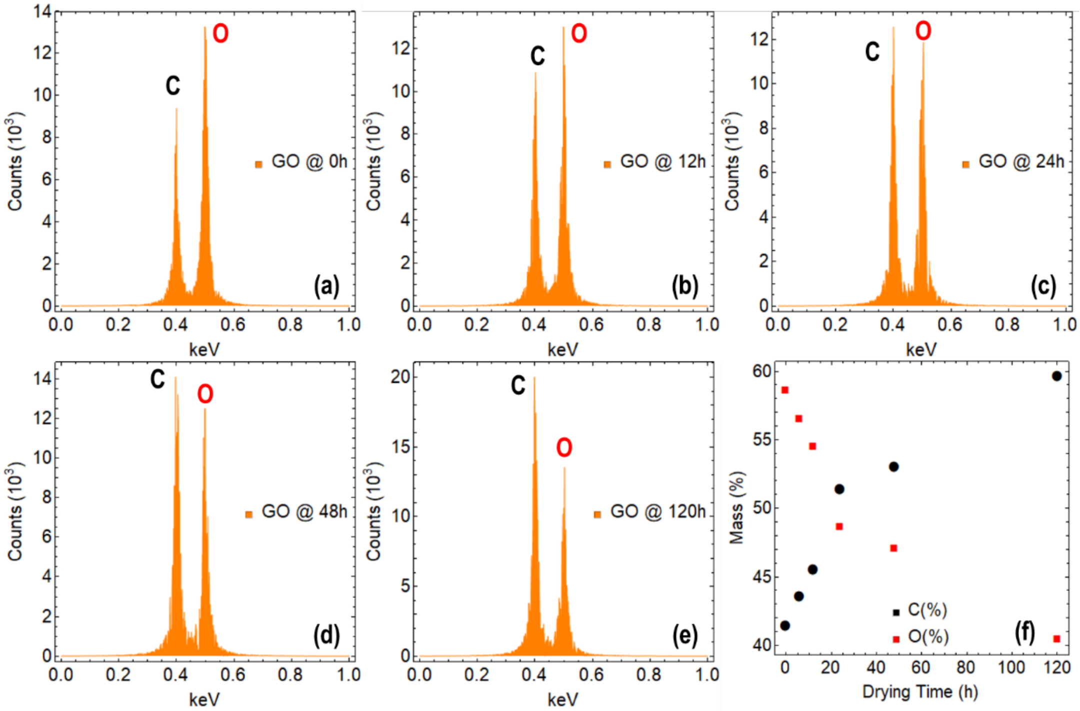

Figure S11. EDS measurements of GO dried at 80 ℃ considering different drying times: (a) 0h, (b) 12 h, (c) 24h, (d) 48, and (e) 120h. (f) Variation of the elemental composition as a function of drying time.

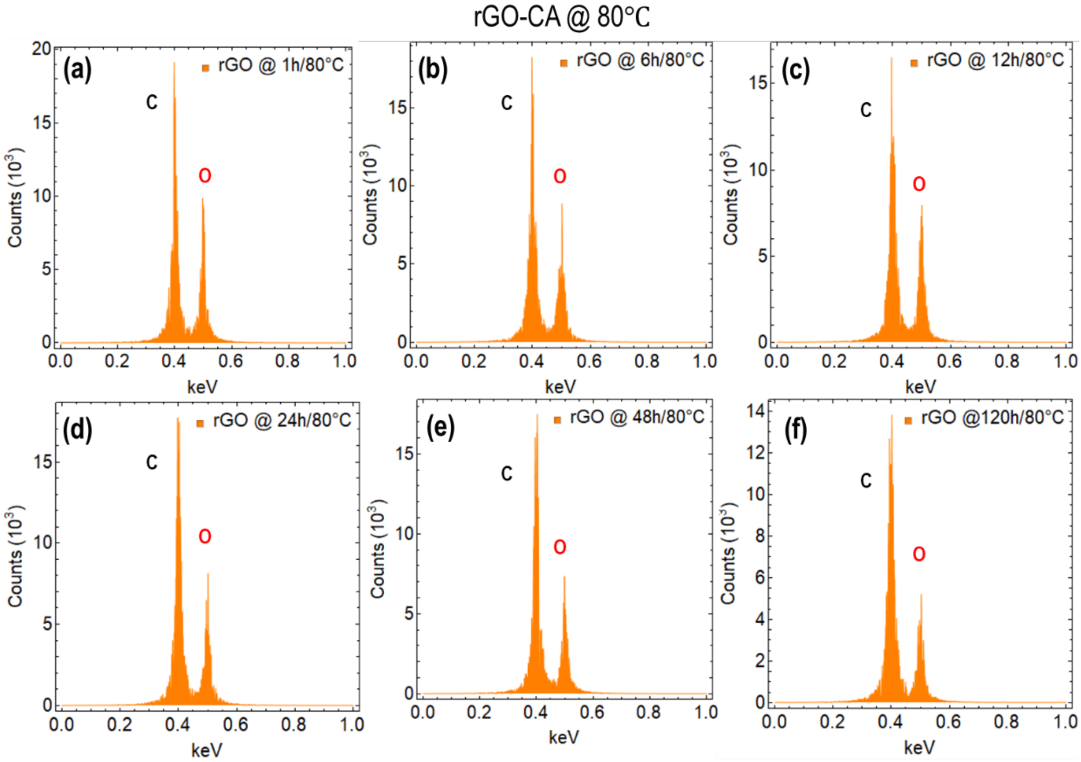

Figure S12. EDS measurements of rGO reduced at 80 ℃ considering different reduction times: (a) 0h, (b) 6h, (c) 12 h, (d) 24h, (e) 48, and (f) 120h.

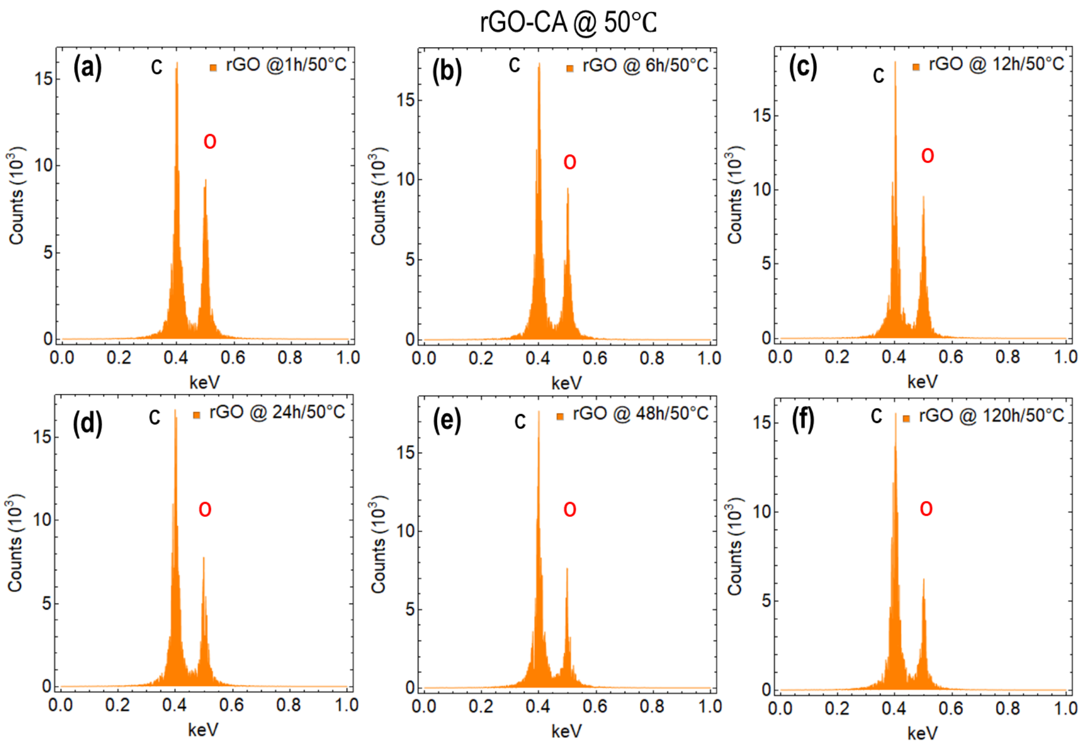

Figure S13. EDS measurements of rGO reduced at 50 °C considering different reduction times: (a) 0h, (b) 6h, (c) 12 h, (d) 24h, (e) 48, and (f) 120h.

Table S1. Peak Position of $\pi - \pi^*$ and $n - \pi^*$transitions in GO dried at 80 ℃ and related full width at half maximums (FWHM), considering different drying times.

Drying Time (h)	$\pi - \pi^*$ Transition (nm)	$n - \pi^*$ Transition (nm)	FWHM (nm)	R^2
0	229.63	305.15	126.57	0.982
6	230.59	305.15	163.92	0.987
12	231.51	305.16	215.92	0.992
24	233.23	305.17	261.59	0.994
48	238.23	305.19	301.72	0.995
120	243.81	305.27	343.90	0.996

Table S2. Estimated optical bandgap values of GO dried at 80 ℃ as a function of drying time.

Drying Time (h)	Optical Bandgap (eV)	R^2
0	4.09	0.996
6	3.82	0.996
12	3.48	0.998
24	3.18	0.998
48	2.94	0.998
120	2.76	0.999

Table S3. Optical absorption coefficient of GO dried at 80 ℃ estimated by a linear fit of the optical absorbance over cell length as a function of concentration under three drying times.

Material	Absorption coefficient (ml mg^{-1} m^{-1})	R^2
GO @ 0 h	3932.22	0.992
GO @ 48 h	4586.71	0.992
GO @ 120 h	5507.15	0.985

Table S4. Peak Position of the $\pi - \pi^*$ transition in rGO reduced at 80 ℃ and related full width at half maximums (FWHM), considering different reduction times.

Drying Time (h)	$\pi - \pi^*$ Transition (nm)	FWHM (nm)	R^2
1	261.73	271.57	0.988
6	262.75	278.39	0.988
12	263.77	311.28	0.989
24	265.81	344.24	0.990
48	269.89	375.28	0.990
120	273.94	406.23	0.991

Table S5. Peak Position of the $\pi - \pi^*$ transition in rGO reduced at 50 ℃ and related full width at half maximums (FWHM), considering different reduction times.

Drying Time (h)	$\pi - \pi^*$ Transition (nm)	FWHM (nm)	R^2
1	260.33	208.94	0.999
6	260.78	221.31	0.999
12	261.56	234.23	0.999
24	263.12	247.03	0.999
48	266.24	259.03	0.999
120	269.36	271.41	0.999

Table S6. Elemental composition of graphite and GO dried at 80 ℃, considering different drying times.

**Sample**	**C (%)**	**O (%)**
Graphite	99.61	---
GO @ 0 h	41.47	58.53
GO @ 6 h	43.75	56.25
GO @ 12 h	45.56	54.44
GO @ 24 h	51.42	48.58
GO @ 48 h	53.01	46.99
GO @ 120 h	59.65	40.35

Table S7. Elemental composition of rGO reduced at 80 ℃, considering different reduction times.

**Sample**	**C (%)**	**O (%)**
rGO @ 1 h	65.99	34.01
rGO @ 6 h	67.28	32.72
rGO @ 12 h	67.52	32.48
rGO @ 24 h	68.53	31.47
rGO @ 48 h	70.44	29.56
rGO @ 120 h	72.68	27.32

Table S8. Elemental composition of rGO reduced at 50 ℃, considering different reduction times.

**Sample**	**C (%)**	**O (%)**
rGO @ 1 h	63.42	36.58
rGO @ 6 h	64.59	35.41
rGO @ 12 h	66.06	33.94
rGO @ 24 h	68.17	31.83
rGO @ 48 h	69.83	30.17
rGO @ 120 h	71.33	28.67
